# The preoperative stratification of patients based on renal scan data is unable to predict the functional outcome after partial nephrectomy

**DOI:** 10.1590/S1677-5538.IBJU.2017.0636

**Published:** 2018

**Authors:** Riccardo Bertolo, Cristian Fiori, Federico Piramide, Daniele Amparore, Francesco Porpiglia

**Affiliations:** 1 Division of Urology, University of Turin, San Luigi Hospital, Orbassano, Turin, Italy; 2 Department of Oncology, University of Turin, San Luigi Hospital, Orbassano, Turin, Italy

**Keywords:** Acute Kidney Injury, Nephrectomy, Carcinoma, Renal Cell

## Abstract

**Introduction:**

eGFR-categories are used to predict functional outcome after partial nephrectomy (PN); no study categorized patients according to preoperative renal scan (RS) data. Aim of the study was to evaluate if stratification of patients according to RS is a reliable method to predict minor/major loss of renal function after PN.

**Materials and Methods:**

We considered patients who underwent PN and RS pre-/post-PN for T1 tumor in our Institution (2007-2017). Demographics, perioperative and specifically functional data were analysed. On the basis of the baseline Split Renal Function (SRF), patients were stratified into risk-categories: 1) baseline operated-kidney SRF range 45-55%; 2) baseline operated-kidney SRF <45%. Risk categories were analysed with postoperative functional outcome: postoperative operated-kidney SRF decrease below 90% of baseline was considered significant loss of function. Contingency tables and univariate/multivariate regression were analysed looking for independent factors of postoperative functional impairment.

**Results:**

224 patients were analysed, 125 (55.8%) maintained >90% of their baseline function. Worse probability of maintaining ≥90 baseline renal function was found in patients with Charlson's Comorbidity Index (CCI≥3) (p=0.004) and patients with PADUA score ≥8 (p=0.023). After stratification by baseline renal function, ischemia was the only independent factor: no effect on patients with poorer baseline renal function. Patients with baseline SRF 45-55% who did not experience ischemia had the highest probability to maintain ≥90% baseline SRF (p=0.028). Ischemia >25 minutes was detrimental (p=0.017).

**Conclusions:**

Stratification of patients by SRF before PN is not a reliable predictor of renal functional outcome. Ischemia seems to scarcely influence patients with poorer renal function.

## INTRODUCTION

Preservation of the maximum amount of operated kidney renal function is the main goal of partial nephrectomy (PN) if compared to radical (1). The majority of the published studies aimed to the report of the functional outcomes after PN have used the estimated Glomerular Filtration Rate (eGFR) as a surrogate measure of the renal function (2, 3). Even if the use of eGFR is easy and cheap, it lacks in accuracy, as it does not take in consideration the compensation by the contralateral kidney (4-6). On the other side, a limited number of studies adopted a more precise method to assess operated kidney function (7). With the aim of assessing the degree of nephron loss after PN and identifying contributing factors, some researches have studied the individual renal unit by using nuclear renal scans (8-13). Indeed, the outcomes of published studies confirmed the role of renal scanning in quantifying the functional loss. By the moment, none of them used renal scanning to classify patients into risk categories on the basis of renal scanning outcomes at pre-operative assessment.

The primary aim of the study was to determine if the stratification of patients according to SRF as assessed by renal scan is a reliable method to classify risk categories for minor or major loss of renal function after PN in comparison to the standard classification according to eGFR into chronic kidney disease stages.

The secondary aim was to look for eventual risk factors for better or worse renal functional outcome on the basis of the created risk categories.

## MATERIALS AND METHODS

### Study Population

We retrospectively reviewed our database dedicated to minimally-invasive nephron sparing surgery and we extracted data regarding all patients who underwent PN between 2007 and 2017. The protocol for the research project was approved by the Institutional Ethics Committee, according to the Declaration of Helsinki.

### Inclusion Criteria

Patients who underwent minimally-invasive PN for cT1 renal mass (14) who had complete data on about the following: 1) evaluation with serum creatinine (SCr), 2) eGFR, as calculated by MDRD (Modification of Diet in Renal Disease) formula (15) and 3) nuclear renal scan (performed in our Institution) both at preoperative assessment and at the third month follow-up.

### Exclusion criteria

Missing data, including Nuclear Renal Scan performed beyond the third month follow-up. Patients who were found to have single kidney or a horseshoe-shaped kidney or renal parenchymal scars at preoperative contrast-enhanced Computed Tomography scan. Patients who experienced complications and/or management of complications potentially impacting on renal function and/or renal volume, such as severe hypotension caused by massive bleeding, embolization or kidney infections.

### Measurements

For each patient extracted from our prospectively maintained database, the following variables were available: demographic variables (including age, gender, body mass index (BMI) and comorbidities, as classified by Charlson's comorbidity index (CCI) (16)); preoperative variables (including the American Society of Anaesthesiologists score, the side, the location, the clinical size and the tumor PADUA score (17) at the preoperative contrast-enhanced Computed Tomography scan); peri-operative data (including the operative time, the management of the renal pedicle, the eventual warm ischemia time (WIT), the estimated blood losses and the intra-operative complications); pathological data (including the final histology, the positive surgical margins rate and the average thickness of the peri-tumoral healthy parenchyma excised); postoperative data (including the postoperative complications as classified by the modified Clavien system (18)).

### Surgical Intervention and Experience

An experienced laparoscopic surgeon (with more than 300 procedures carried out at the beginning of the considered time span) performed the key steps of all the surgeries (tumour resection and renorrhaphy), according to a previously described technique (19). No dedicated anaesthetic procedures (such as controlled hypotension) were used. Renorrhaphy was performed in all cases dedicated running suture of the kidney medulla and cortex, secured by Absolok^®^ (Ethicon Endo-Surgery, Inc., Cincinnati, OH, USA) and Hem-o-lok^®^ clips (Weck Surgical Instruments, Teleflex Medical, Durham, NC, USA), respectively (20).

### Functional Evaluation

Specifically for the purpose of the study, all patients had undergone evaluation of renal function with SCr, eGFR (according to the MDRD formula) and Split Renal Function (SRF) calculated as the percentage of contribution to overall renal function by the operated kidney by mean of the nuclear renal scan. All the examinations were performed preoperatively and at the third month postoperatively. Tc-99m mercapto-acetyl triglycine 3 renal scan was performed in all cases. All renal scans were performed at our Institution and read by a dedicated nuclear medicine doctor.

On the basis of the baseline assessment of SRF, patients were stratified into two risk categories. Risk category 1 included all patients with baseline operated kidney SRF ranging from 45 to 55%; risk category 2 included all patients with baseline operated kidney SRF <45%.

The criterion was arbitrary but based on institutional expert opinion (21).

Risk categories were compared on the basis of the postoperative functional outcome: risk category migration and the percentage of maintenance of operated kidney baseline renal function were both considered in the analysis. Operated kidney postoperative SRF decrease below 90% of its baseline was considered as significant loss of renal function (i.e from baseline SRF=45 to postoperative SRF=40 represented a significant decrease of operated kidney baseline SRF because equal to 11.1% decrease).

### Pathology Assessment

A dedicated uro-pathologist analysed fresh-tissue specimens from the operating room and defined primary tumour extent in accordance with TNM classification (14). A mean value for peri-tumor healthy tissue thickness was obtained (22).

### Statistical methods

Patient's characteristics were tested by the Fisher's exact test for categorical variables and by the Mann-Whitney and Wilcoxon tests for continuous ones. All results for the continuous variables were expressed as the mean and the standard deviation; all the results for the categorical variables were expressed as the median and the inter-quartile range (IQR). Different contingency tables were presented, in order to analyse the influence of unmodified patient variables either on preoperative or postoperative SRF. The univariate/multivariate binary logistic regression model was used to test age (>65 vs. ≤65 yrs), gender (male vs. female), BMI (>25 vs. ≤25), Charlson Index (≥3+ vs. <3), PADUA score (≥8 vs. <8), GFR (<60 vs. 60-90 vs. ≥90 mL/min.), blood loss (<150 vs. ≤150 mL), warm ischemia time (>25 vs. ≤25 min vs. no ischemia) and average tissue of perilesional healthy parenchyma excised (>2.65 vs. ≤2.65 mm) (independent variables) as risk factors for an SRFpostoperatory / SRFpreoperatory ratio <90% versus ≥90% (dependent variable). The median value of the distribution for every tested variable was chosen as the cut-off. All reported p-values were obtained by the two-sided exact method, at the conventional 5% significance level. Data were analysed by R 3.2.1 (https://www.r-project.org)

## RESULTS

Two-hundred-twenty-four patients were considered in the analysis.

Patient's demographics and renal nephrometric features are reported in Table-1. Eighty-eight patients (39.3%) were over 65 years old. One hundred-thirty-seven patients (61.2%) had BMI over 25. Ninety-five patients (42.4%) had Charlson Comorbidity Index ≥3. One hundred-forty-seven patients (65.6%) had PADUA score ≥8.

**Table 1:** Patients’ demographics and lesions characteristics.

Variables	Result
No. patients	224
Males, No. (%)	161 (71.8)
Age, years, mean (SD)	60.5 (11.4)
No. Age > 65 (%)	88 (42.4)
BMI (kg/m^2^), mean (SD)	24.2 (5.6)
No. BMI > 25 (%)	137 (61.2)
CCI, median (IQR)	1 (0-2)
CCI Age-Adjusted, median (IQR)	2 (2-3)
No. CCI ≥ 3 (%)	95 (42.4)
ECOG PS, median (IQR)	0 (0-1)
ASA score, median (IQR)	1 (1-1)
Mean preop.ve Hb (SD), mg/dL	13.5 (2.4)
Mean CT-scan lesion size (SD), mm	50.8 (16.1)
No. right-sided tumors (%)	108 (48.2)
PADUA Score, median (IQR)	10 (9-11)
No. PADUA score ≥ 8 (%)	147 (65.6)

**SD** = Standard Deviation; **BMI** = Body Mass Index; **CCI** = Charlson's Comorbidity Index; **IQR** = Inter-Quartile Range; **ECOG PS** = Eastern Cooperative Oncology Group Performance Status; **ASA** = American Society of Anaesthesiologists; **Hb** = Haemoglobin; **CT** = Computed Tomography; **PADUA** = Preoperative Aspects and Dimensions Used for Anatomical.

Patient's baseline renal function parameters are reported in Table-2. Eighty-three (37.1%), 110 (49.1%) and 31 (13.8%) patients had baseline eGFR >90, ranging from 60 to 90 and <60 mL/min., respectively.

**Table 2:** Renal function data.

Variables		Result
Preoperative SCr (mg/dL), mean SD		1.04 (0.32)
Preoperative eGFR (mL/min.), mean SD		75.7 (23.27)
– MDRD formula		
No. patients with baseline eGFR (%)		
≥ 90		67 (29.9)
≥ 60,< 90		105 (46.9)
< 60		52 (23.2)
Preoperative Split Renal Function, mean SD		47.1 (7.6)
**Split Renal function Risk category at baseline– No. patients (%)**		
	45-55	166 (74.1)
	< 45	58 (25.9)
Postoperative SCr (mg/dL), mean SD		1.19 (0.46)
Postoperative eGFR (mL/min.), mean SD – MDRD formula		68.8 (26.22)
% Δ Scr (preoperative vs postoperative)		+14.8 (25.97)
% Δ eGFR (preoperative vs postoperative)		-12.4 (20.1)
Postoperative Split Renal Function, mean SD		44.5 (8.9)
% Δ Split Renal Function (preoperative vs postoperative)		-10.21 (8.6)

**Scr** = Serum Creatinine; **SD** = Standard Deviation; **eGFR** = estimated Glomerular Filtration Rate; **MDRD** = Modification of Diet in Renal Disease.

**Figure 1:**
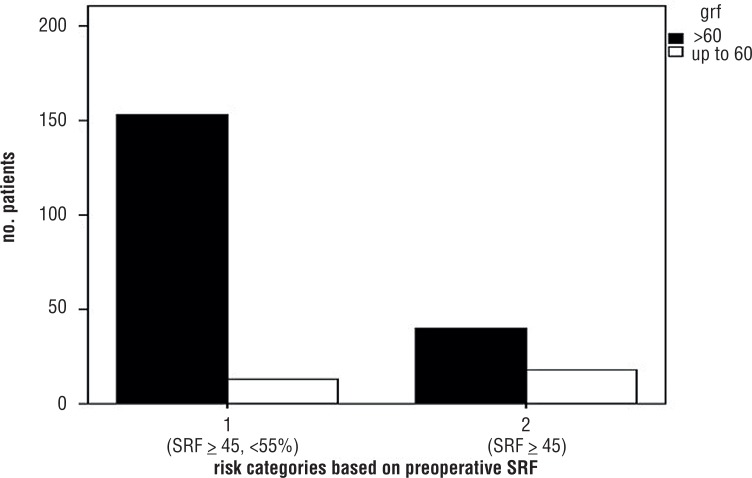
Bar chart depicting patients stratified into the risk categories based on preoperative Split Renal function: on the right, risk category 1 (patients with baseline split renal function at renal scan ranging between 45 and 55%); on the left, risk category 2 (patients with baseline split renal function at renal scan < 45%); patients with baseline estimated Glomerular filtration Rate > 60 mL/min. are represented in the blue bars; patients with baseline estimated Glomerular filtration Rate ≤ 60 mL/min. are represented in the green bars, 92.2% of patients with baseline split renal function at renal scan ranging between 45 and 55% had Glomerular filtration Rate > 60 mL/min.

One hundred-sixty-six patients (74.1%) had baseline Split Renal Function as estimated by renal scan ranging from 45 to 55%. High association between SRF ranging from 45 to 55% eGFR ≥60 mL/min. was found (92.2%, respectively - Figure-1).

Patient's perioperative data are reported in Table-3. One hundred-four (46.4%), 95 (42.4%) and 25 (11.2%) patients underwent 0, ≤25 and >25 minutes of warm ischemia, respectively.

**Table 3:** Perioperative variables.

Variables		Result
**No. management of renal artery (%)**		
	• Clampless (0 min ischemia)	99 (44.2%)
	• Global ischemia ≤ 25 min	90 (40.2%)
	• Global ischemia > 25 min	35 (15.6%)
Mean WIT (SD), min.		19.6 (5.7)
Mean EBL (SD), mL		131.5 (226.8)
Mean operative time (SD), min		112.1 (33.2)
No. intraoperative complications (%)		2 (< 1.0)
No. postoperative complications (%)		27 (12.0)
No. Clavien grade ≥ III (%) postoperative complications		6 (2.7)
Median hospital stay (IQR), days		5 (4–6)

**WIT** = Warm Ischemia Time; **SD** = Standard Deviation; **EBL** = Estimated Blood Losses; **IQR** = Inter-Quartile Range

Concerning pathological data, 188 patients (83.9%) were found to have renal cancer. Mean tumor size was 48.6±15.3 mm. Among patients with malignancies, five patients (2.6%) had positive surgical margins. Mean thickness of peri-tumoral healthy tissue excised was 2.8±1.7 mm.

Postoperative functional outcomes are reported in Table-2 again. As expected, the median operated kidney postoperative SRF was higher in patients classified in the risk category 1 at baseline (p=0.003).

Overall, 125 patients (55.8%) maintained ≥90% of their baseline renal function.

Concerning contingency tables, patients with PADUA score <8 were more likely to maintain their postoperative renal function ≥90% (p=0.023). Patients who underwent clampless PN were more likely to maintain their postoperative renal function ≥90% (p <0.001). Patients who underwent WIT >25 minutes were more likely to have postoperative renal function <90% of their baseline in 72.0% of cases. The worst probability of maintaining ≥90 baseline renal function was found in patients with CCI ≥3 (15.0%, p=0.004). Univariate logistic regression analysis (see Table-4) confirmed CCI, PADUA score and WIT >25 minutes as risk factors for postoperative loss of renal function (postoperative SRF <90% of the baseline value at postoperative control). At multivariate logistic regression analysis (Table-4) WIT was confirmed as independent variable.

**Table 4:** Univariate and Multivariate Logistic Regression Models.

Univariate	Outcome: < 90% SRF maintained	OR	95% C.I.	*p-value*
Age, years	> 65 vs. ≤ 65	1.59	0.93–2.73	0.093
Gender	Female vs Male	0.85	0.48–1.50	0.569
BMI, kg/m^2^	> 25 vs. ≤ 25	1.21	0.70–2.08	0.499
CCI	≥ 3 vs. < 3	1.68	1.12–2.88	0.032
PADUA score	≥ 8 vs. < 8	1.94	1.09–3.43	0.024
eGFR, mL/min. x 1.73 m^2^	60-90 vs. > 90	1.14	0.64–2.03	0.649
	< 60 vs. > 90	1.13	0.49–2.59	0.774
WIT, min				< 0.001
	≤ 25 vs. 0 ischemia	2.40	1.34–4.28	0.003
	> 25 vs. 0 ischemia	5.79	2.20–15.22	< 0.001
EBL, mL	> 150 ≤ 150	1.10	0.65–1.86	0.733
Healthy margin excised, mm	> 2.65 vs. ≤ 2.65	0.87	0.48–1.57	0.640
Multivariate	Outcome: < 90% SRF maintained	OR	95% C.I.	*p-value*
CCI	≥ 3 vs. < 3	1.45	0.76–2.77	0.256
PADUA score	≥ 8 vs. < 8	1.62	0.89–2.98	0.117
WIT, min.				< 0.001
	≤ 25 vs. 0 ischemia	2.42	1.35–4.34	0.003
	> 25 vs. 0 ischemia	6.58	2.46–17.62	< 0.001

**SRF** = Split Renal Function; **OR** = Odd Ratio; CI: Confidence Interval; **BMI** = Body Mass Index; **CCI** = Charlson's Comorbidity Index; **PADUA** = Preoperative Aspects and Dimensions Used for Anatomical; **eGFR** = estimated Glomerular Filtration Rate; **WIT** = Warm Ischemia Time; **EBL** = Estimated Blood Losses.

At separate evaluation of the risk categories after stratification, 94.8% of patients classified in risk category 2 at baseline were confirmed in risk category 2 at the postoperative assessment.

**Figure 2:**
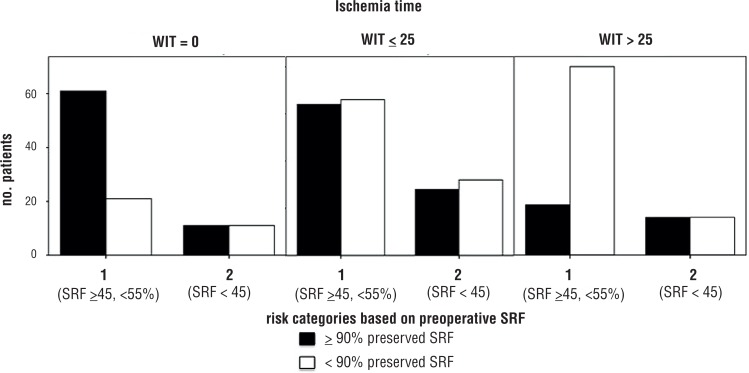
Bar charts depicting patients stratified according to the warm ischemia time. In every chart on the right risk category 1 patients (with baseline split renal function at renal scan ranging between 45 and 55%); on the left risk category 2 patients (with baseline split renal function at renal scan < 45%); patients with preservation of ≥ 90% of the baseline Split Renal function are represented in the blue bars; patients with preservation of < 90% of the baseline Split Renal function are represented in the green bars.

Patients with baseline SRF ranging from 45 to 55% who did not experienced renal ischemia (the so called “clampless” PN) had a higher probability to maintain ≥90% of their baseline renal function (74.4%, p=0.028). Conversely, patients in risk category 1 who experienced ischemia time over 25 minutes had a worse outcome (78.9%, p=0.017, Figure-2).

## DISCUSSION

The results of the present study showed that the classification of patients by SRF does not seem to be a reliable method to preoperative differentiate patients into risk categories influencing operated kidney functional outcome. Indeed, 55.8% of patients maintained >90% of their baseline renal function, regardless the preoperative risk category they started.

On the other side, we found that renal scan data were confirmed by the “standard” renal function parameters: indeed, more than 90% of patients with baseline operated kidney SRF ranging from 45 to 55% (risk category 1) had both SCr <1.2 and eGFR >60 mL/min., confirming the normal baseline renal function.

Concerning the potential predictors of postoperative functional outcome, patients with PADUA score ≥8, or who underwent warm ischemia over 25 minutes, or who had CCI ≥3 were less likely to maintain >90% of their baseline SRF. These findings were found regardless the risk category.

Surgical ischemia was the only factor showing different effects in the two risk categories: indeed, no significant effects were found in the risk category 2 (patients with preoperative SRF <45%), whilst, in category 1 (patients with preoperative SRF ranging from 45 to 55%), patients who avoided ischemia were more likely to maintain their baseline SRF >90%; on the contrary, patients who had ischemia over 25 minutes had worsened outcome.

Some considerations about the findings of the present study are required.

The range commonly used by the nuclear medicine literature to describe a “normally” functioning kidney (21) revealed to be correct as we found in our study a more than 90% of concordance of set range for SRF with normal SCr and eGFR.

Specifically regarding the primary aim of the study, the classification of the patients by risk categories revealed to have scarce power in predicting the operated kidney functional outcome after PN. Indeed, the power of the risk categories in predicting the probability of experiencing a significant loss of renal function was equal to flipping a coin.

Contingency tables revealed that CCI ≥3 plays a negative effect so that 15% only of patients with such feature maintained more than 90% of their baseline SRF, confirming the current knowledge about comorbidities role in influencing the postoperative functional outcomes: it is known that loss of renal function after PN is a multifactorial process related to both modifiable factors (duration of ischemia and removal of unaffected nephrons) and unmodifiable factors (age, comorbidities and pre-operative renal function) (23, 24).

Again, confirming literature data, PADUA score ≥8 (indicating more moderate to high complexity lesions) was found to be a predictor of worse probability to maintain baseline SRF (25). Intuitively, the finding showed that more complex renal lesions could affect renal functional outcome. It could be due to the wider amount of renal parenchyma resected during PN or due to the more complex suture demanded after complex resection.

We underline that the above-mentioned findings were true regardless the risk category at baseline.

The significant finding after stratification according to the described risk group categories regarded the surgical renal ischemia. Particularly, if no effect of ischemia versus no ischemia was found in the patients with worse renal function at baseline (in risk category 2), conversely, patients starting from risk category 1 (SRF ranging from 45 to 55% at preoperative assessment) were more influenced by ischemia, both in the case of prolonged and avoided ischemia: in fact, prolonged ischemia, over the described critical threshold of 25 minutes (2, 24) was found to have detrimental effect on the patients of the studied cohort, with only 20% of these patients maintaining >90% of their baseline renal function.

On the other side, around 75% of patients with superior operated kidney baseline function who underwent clampless PN maintained >90% of baseline renal function. In summary, it seemed that avoiding the clamping of renal artery was protective from postoperative decrease in renal function: the “unusual” was not to record this trend in patients with worse baseline function.

Indeed, previous reports were contradictory with respect to finding of the present study as stating that a trend towards a major benefit in postoperative renal function by avoided clamping of renal artery could be observed in patients with poorer baseline renal function (26-28).

In the present paper, written by analysing a larger sample size, the “novel” finding would state that a “normal” kidney with avoided ischemia represents the best condition. Maybe the healthy kidney takes more advantages by the avoided at all ischemia. It is possible that some conditions underlying in the altered renal function in case of preoperative renal disease are able to eliminate the positive effect of avoided ischemia. Moreover, we can state that the kidney with worse baseline renal function has much less to loose after the intervention, whatever the management of the renal pedicle and the ischemia.

Using total eGFR tends to overestimate the degree of renal function preservation after PN, and this is particularly relevant when studying factors affecting functional outcomes after nephron-sparing surgery like in the cases reported herein. Ipsilateral renal function is a more precise assessment method in this setting as previously described (29).

The study was not devoid of limitations, principally related to the retrospective nature and to the sample size considered. As per retrospective design studies, a selection bias could affect the results.

Notwithstanding the limitations, we underline that the rigorous selection of patients who underwent renal scan preoperatively and at the third month postoperatively in the institutional division of nuclear medicine surely increased the value of the data but it reduced the sample size at the analysis.

Further studies, ideally prospective, with a larger sample size would be needed in order to confirm our reports.

## CONCLUSIONS

The stratification of patients by SRF as assessed by renal scan before PN does not seem to work as a valuable tool for predicting the postoperative renal functional outcome after the intervention. Lesion's complexity, ischemia time and comorbidities are confirmed to play a role in determining the postoperative functional outcome, regardless the baseline renal function.

Ischemia time seems to have scarce effects on patients with poor baseline renal function maybe because they have much less to loose. No ischemia has a positive effect on patients with normal baseline renal function. The same patients were found to suffer more from a prolonged ischemia.
